# Proximity Labelling‐Based Proteomics Identifies Antiviral Host Factors Associated With the Potexvirus Replicase

**DOI:** 10.1111/mpp.70239

**Published:** 2026-03-19

**Authors:** Yi‐Chen Chang, Sae Takatsuka, Aki Eguchi, Nobumitsu Sasaki, Yoshiyuki Itoh, Miki Hisada, Tsutomu Arie, Ken Komatsu

**Affiliations:** ^1^ United Graduate School of Agricultural Science Tokyo University of Agriculture and Technology (TUAT) Fuchu Japan; ^2^ Graduate School of Agriculture Tokyo University of Agriculture and Technology (TUAT) Fuchu Japan; ^3^ Institution of Global Innovation Research (GIR) Tokyo University of Agriculture and Technology (TUAT) Fuchu Japan; ^4^ Smart‐Core‐Facility Promotion Organization Tokyo University of Agriculture and Technology (TUAT) Fuchu Japan

**Keywords:** endoplasmic reticulum, membrane contact sites, Plantago asiatica mosaic virus, proximity labelling, viral replication complex

## Abstract

Plant positive‐strand RNA viruses establish viral replication complexes (VRCs) by remodelling intracellular membranes, primarily the endoplasmic reticulum (ER) and recruiting host proteins to these structures. To identify ER‐associated host proteins involved in potexvirus replication, we developed a multi‐control proximity labelling approach using TurboID‐fused constructs that localise to the cytosol, the ER lumen and the ER membrane. This strategy enabled specific identification of host factors that are proximal to the methyltransferase (MET) domain of Plantago asiatica mosaic virus (PlAMV) replicase, distinguishing them from general ER‐associated proteins. Our proximity labelling identified 33 host factors associated with the PlAMV MET domain, 28 of which showed exclusive enrichment compared to all three controls. Subcellular localization prediction revealed that MET‐proximal proteins predominantly associate with chloroplasts, the cytosol and mitochondria rather than the ER. This suggests that the MET domain localises to specialised ER membrane subdomains interfacing with multiple organelles. Functional analysis of three selected candidates, calcyclin‐binding protein (NbCBP), remorin1.5 (NbREM1.5) and calcium‐sensing receptor (NbCAS), revealed their antiviral roles. Virus‐induced gene silencing of these factors significantly enhanced PlAMV accumulation. Co‐immunoprecipitation demonstrated physical interactions between the three host factors and the MET domain, while confocal microscopy revealed their relocalization to specific subcellular sites during co‐expression with MET. These results demonstrate that potexvirus VRCs interact with diverse host factors from multiple organelles, providing new insights into plant–virus interaction networks.

## Graphical abstract

Multi‐control proximity labelling identifies host factors at ER‐organelle interfaces. NbCBP, NbREM1.5 and NbCAS interact with the PlAMV MET domain and restrict viral replication.

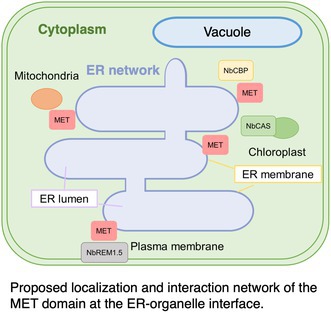

## 1 Introduction

Positive‐strand RNA ([+]RNA) viruses represent the most prevalent type of plant viruses, causing diverse diseases in agriculturally important crops. These viruses establish viral replication complexes (VRCs) or organelles (VROs) by extensively remodelling intracellular membranes (Miller and Krijnse‐Locker 2008; Romero‐Brey and Bartenschlager 2016; Tao and Ye 2010). Among the intracellular membranes, the endoplasmic reticulum (ER) serves as the primary site for replication of many plant (+)RNA viruses. The ER, which participates in cellular processes including lipid synthesis, membrane curvature and the regulation of the cellular redox environment (Nishikiori and Ahlquist 2018), provides a physical scaffold for VRC assembly. This can create an environment enriched with host factors essential for viral replication, and also provide protection of viral genomes against antiviral host defences (Alberts et al. 2002; English and Voeltz 2013). In addition, recent studies have demonstrated that the ER serves as a battleground between plants and RNA viruses, where antiviral defence responses, including RNA silencing, operate through interaction with other intracellular organelles (Adhikari et al. 2025; Huang et al. 2025). Despite the critical importance of understanding virus–host interactions at the ER, comprehensive knowledge of the host factors involved in VRC formation or those targeting VRCs to inhibit viral infection remains limited.

Plantago asiatica mosaic virus (PlAMV) is a (+)RNA virus belonging to the genus *Potexvirus*, with a genome containing five open reading frames encoding a replicase, triple gene block proteins and a coat protein (Komatsu and Hammond 2022). The replicase is composed of three domains: methyltransferase (MET), helicase and replicase domains. Among these, the MET domain appears to play a foundational role in establishing the replication environment. We previously identified a membrane‐associated amphipathic α‐helix within the MET domain of the PlAMV replicase. In the same study, we found through transient expression analysis that the MET domain fused with green fluorescent protein (MET‐GFP) alone is sufficient to induce localization in close proximity to the ER, where it forms discrete granules in the cell periphery (Komatsu et al. 2021). These observations are consistent with our recent work using an antibody‐based probe, which confirmed that the full‐length replicase targets the same ER‐proximal sites during actual PlAMV infection, consistent with the localization pattern of the MET‐GFP (Ishihara et al. 2025). While the large complex‐forming ability of the MET domain is essential for VRC establishment, its specific interactions with the host cellular machinery remain to be fully elucidated. Given that interactions between viral auxiliary proteins and host factors are critical for organising the VRC, the MET domain likely acts as a strategic platform to recruit host factors necessary for replication. In the preceding co‐immunoprecipitation and mass spectrometry (Co‐IP/MS) studies employing the MET domain as a bait, a dynamin‐like protein was identified as a proviral factor for PlAMV replication (Shinji et al. 2023). To achieve a comprehensive understanding of the proviral and antiviral host factor repertoire specifically involved in the early organisation and function of the PlAMV replication complex, we used the MET domain as the primary bait for our proximity labelling analysis.

Proximity labelling (PL), which utilises biotin ligases such as TurboID to label proximal proteins, has emerged as a powerful technique for studying protein–protein interactions by enabling target labelling without disrupting cellular structures (Branon et al. 2018). This approach effectively captures transient interactions and characterises subcellular organelle proteomes in vivo (Antonicka et al. 2020; Gingras et al. 2019; Hung et al. 2017), with successful applications in plant systems including the analysis of the nuclear envelope, chloroplast, mitochondrial and peroxisomal proteomes (Bao et al. 2024; Tang et al. 2020). Recently, PL was employed to identify host proteins that interact with the p23 replication protein of the ER‐replicating beet black scorch virus (BBSV), revealing the proviral function of reticulon‐like proteins (Zhang et al. 2023). However, the PL‐mediated identification of host proteins that interact with plant viral replicases is still limited.

In this study, by utilising the PL methodology combined with the multi‐control approach, we investigated the ER‐proximal proteomes involved in PlAMV infection, based on the hypothesis that MET‐mediated VRC formation and antiviral responses targeting this step are mediated by host factors localised in close proximity to the ER (Komatsu et al. 2021). We employed three TurboID‐fused controls: ER membrane‐targeting (2C1‐GFP‐Turbo), ER lumen‐targeting (GFP‐Turbo‐HDEL) and cytosolic‐targeting (GFP‐Turbo) proteins. These controls were used to establish comprehensive baseline proteomes for comparative analysis, enabling the distinction between ER‐proximal host factors and general ER‐associated proteins. Confocal microscopy combined with protein fractionation assays confirmed the proper subcellular localization of each TurboID protein. Comparative analyses involving the TurboID‐fused MET domain, as well as functional analyses, identified three unique host factors that co‐precipitate with MET, undergo subcellular relocalization upon MET co‐expression and exhibit antiviral activity. Our PL approach provides new insights into the subcellular organisation of the PlAMV replicase at the ER and identifies host factors that modulate viral infection.

## 2 Results

### 2.1 TurboID‐Fused MET Domain of PlAMV Replicase Differs in Subcellular Localization and Membrane Association Properties Compared to ER‐Localising TurboID Controls

**FIGURE 1:**
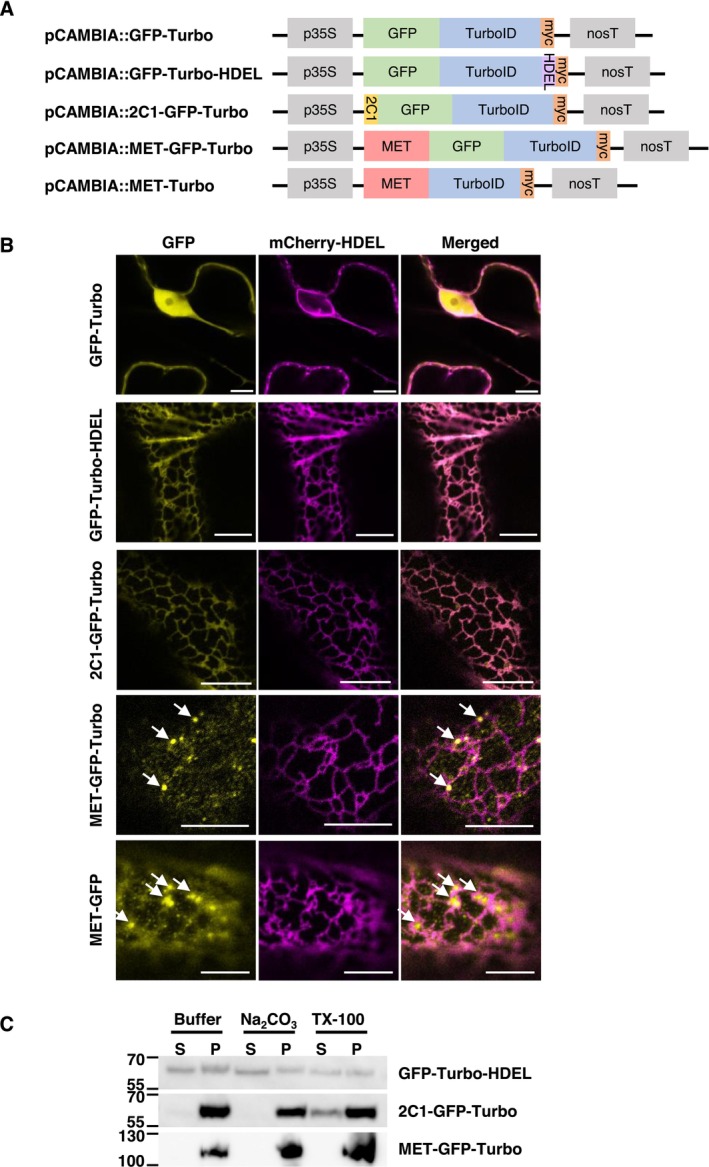
Subcellular localization of TurboID‐fused MET domain of Plantago asiatica mosaic virus (PlAMV) replicase is different from that of two endoplasmic reticulum (ER)‐targeting TurboID proteins. (A) Schematic diagrams of TurboID‐fused constructs. Gene expression was controlled by cauliflower mosaic virus 35S promoter (p35S) and nopaline synthase terminator (nosT). *HDEL*, ER retention signal; *2C1*, the N‐terminal signal anchor of cytochrome P‐450 2C1 that mediates retention in the ER membrane; *MET*, the methyltransferase domain of the replicase of PlAMV; myc, c‐Myc epitope tag. (B) Subcellular localization of GFP‐Turbo, GFP‐Turbo‐HDEL, 2C1‐GFP‐Turbo, MET‐GFP‐Turbo and MET‐GFP. Each TurboID‐fused protein was co‐expressed with mCherry‐HDEL in *Nicotiana benthamiana* by agroinfiltration. Epidermal cells were observed by confocal microscopy at 1 day post‐infiltration (dpi). Arrows indicate the positions of the granules formed by MET‐GFP‐Turbo or MET‐GFP. Scale bars = 10 μm. (C) Immunoblotting analyses of GFP‐Turbo‐HDEL, 2C1‐GFP‐Turbo, or MET‐GFP‐Turbo. The difference in the migration rate of GFP‐Turbo‐HDEL between S and P fractions likely reflects different levels of protein processing or post‐translational modifications. At 1 dpi, protein was extracted, and pellet fraction was obtained by centrifugation at 30,000 *g*, which was treated with buffer (control), Na_2_CO_3_ or Triton X‐100, followed by an additional centrifugation at 30,000*g* to separate soluble (S) and pellet (P) fractions. Immunoblotting was performed using anti‐c‐Myc antibody.

To identify host factors that interact with the MET domain of PlAMV replicase, which associates with the ER network and contributes to VRC formation (Komatsu et al. 2021), we developed PL constructs that produce the biotin ligase, TurboID, fused to the C‐terminus of MET, with or without GFP (MET‐GFP‐Turbo and MET‐Turbo; see Figure 1A). To distinguish MET‐specific ER‐proximity proteins from non‐specific ER‐associated background proteins, three TurboID‐fused GFP controls were used: GFP‐Turbo (without any localization signals), GFP‐Turbo‐HDEL (with the ER retention signal HDEL) and 2C1‐GFP‐Turbo (with the N‐terminal signal anchor of cytochrome P450 2C1; Ahn et al. 1993; Lam et al. 2014), which would target the nucleocytoplasm, the ER lumen and the ER membrane, respectively. All TurboID‐fused proteins were C‐terminally tagged with a c‐Myc epitope and expressed under the control of the cauliflower mosaic virus 35S promoter (p35S) and the nopaline synthase terminator (nosT) and the protein expression and biotinylation efficiency were checked by immunoblotting (Figure 1A; Figure S1).

To confirm that fusion with TurboID does not alter the intrinsic subcellular localization of each protein, TurboID‐fused GFP controls and MET‐GFP‐Turbo were transiently expressed in *Nicotiana benthamiana* leaves using agroinfiltration, and their subcellular localization was examined by confocal microscopy at 1 day post‐infiltration (dpi). This time point was specifically selected to capture the early stages of host–virus interactions relevant to initial VRC assembly. As expected, GFP‐Turbo localised to the cytoplasm and nucleus, which was clearly distinct from the co‐expressed ER marker, mCherry‐HDEL (Figure 1B, top row). In contrast, both GFP‐Turbo‐HDEL and 2C1‐GFP‐Turbo exhibited reticulated network patterns that co‐localised with mCherry‐HDEL (Figure 1B, second and third rows). Meanwhile, MET‐GFP‐Turbo formed small granules closely associated with the ER network (Figure 1B, fourth row), confirming that the TurboID fusion does not disrupt the characteristic subcellular localization of MET‐GFP granules in close proximity to the ER (Figure 1B, bottom row) (Komatsu et al. 2021).

To further characterise the membrane association properties of MET‐GFP‐Turbo and the two ER‐localised GFP‐Turbo proteins, we performed biochemical fractionation and membrane extraction analyses. All three proteins (GFP‐Turbo‐HDEL, 2C1‐GFP‐Turbo and MET‐GFP‐Turbo) partitioned into the membrane‐enriched pellet fraction. The fractions were then treated with either an alkaline buffer (Na_2_CO_3_) or a non‐ionic detergent (Triton X‐100), after which they were subjected to further fractionation. GFP‐Turbo‐HDEL was extracted into the soluble fraction by both Na_2_CO_3_ and Triton X‐100, consistent with its localization within the ER lumen. Conversely, 2C1‐GFP‐Turbo was resistant to Na_2_CO_3_ extraction but was solubilised by Triton X‐100, confirming its status as an integral membrane protein anchored in the ER membrane (Figure 1C). Notably, MET‐GFP‐Turbo remained predominantly in the pellet fraction after treatment with either Na_2_CO_3_ or Triton X‐100 (Figure 1C), suggesting its association with detergent‐resistant membranes (Borner et al. 2005; Mongrand et al. 2004). Taken together, these results confirm that GFP‐Turbo‐HDEL and 2C1‐GFP‐Turbo localise to the ER lumen and ER membrane, respectively, and also suggest that MET associates with the ER membrane in a distinct manner from the two ER marker proteins.

### 2.2 Comparative Proximity Labelling of the ER‐Associated Proteins Using ER Lumen‐ and Membrane‐Localising TurboID Controls

**FIGURE 2:**
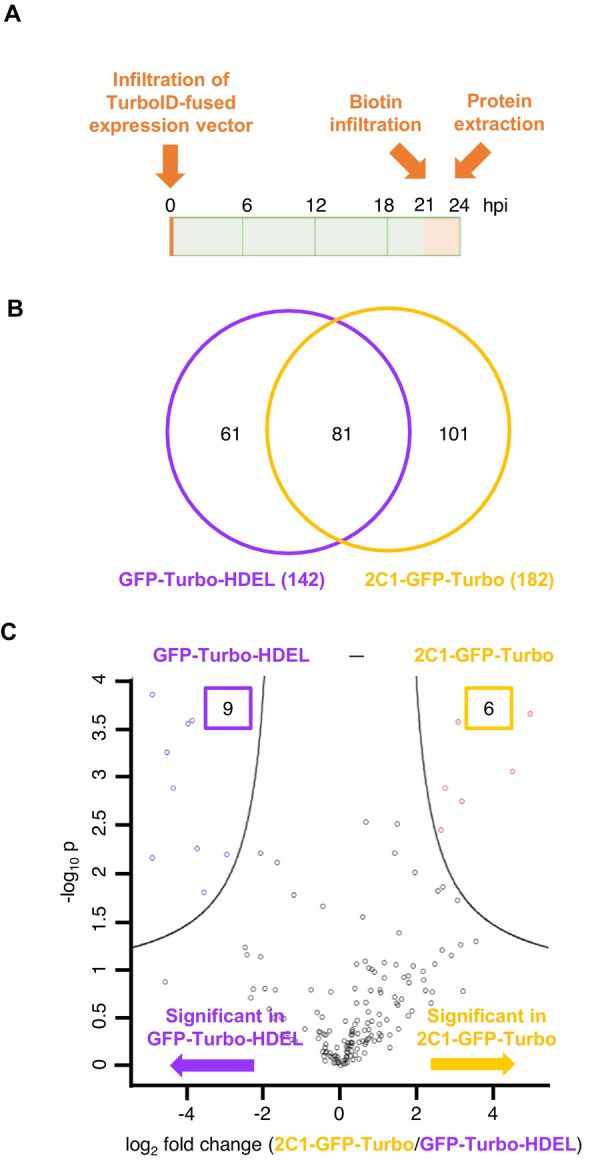
Comparative proximity labelling of endoplasmic reticulum (ER)‐associated proteins using ER lumen‐ and membrane‐localising TurboID controls. (A) Experimental workflow for proximity labelling. Timeline shows time points of biotin (50 μM) treatment and protein extraction relative to that of agroinfiltration (hours post‐infiltration, hpi). (B) Venn diagram showing the number of biotinylated proteins captured by GFP‐Turbo‐HDEL and 2C1‐GFP‐Turbo, with their overlapping proteins from three independent experiments. (C) Volcano plots of pairwise differential protein analysis comparing GFP‐Turbo‐HDEL versus 2C1‐GFP‐Turbo (*n* = 3). Significantly enriched proteins are highlighted in blue or red and separated by curved significance boundaries. Numbers indicate significantly enriched proteins for each construct. Statistical analysis was performed using Perseus with filtering criteria: S_0_ = 1.0 and FDR ≤ 0.05.

To validate whether our ER‐localising TurboID fusions could capture distinct ER‐associated proteins and serve as appropriate controls for MET‐Turbo, we performed PL using GFP‐Turbo‐HDEL and 2C1‐GFP‐Turbo. Recent studies have demonstrated that endogenous biotin levels in plants can support low‐level TurboID‐mediated biotinylation (Feng et al. 2023; Mair et al. 2019), emphasising the importance of optimising both biotin concentration and treatment duration to minimise non‐specific labelling. After a preliminary optimisation experiment, we performed PL according to the timeline shown in Figure 2A, with 50 μM of biotin applied at 21 h post‐infiltration (hpi) with *Agrobacterium* containing each construct in 4‐week‐old *N. benthamiana* leaves. Biotinylated proteins from three independent biological replicates were purified after 3 h of biotin treatment and analysed by LC–MS/MS. Using the *N. benthamiana* NbDE proteome database (Kourelis et al. 2019), 142 and 182 proteins were identified for GFP‐Turbo‐HDEL and for 2C1‐GFP‐Turbo, respectively, with 81 proteins overlapping between the two ER‐localising markers (Figure 2B).

A pairwise comparison identified 9 and 6 significantly enriched proteins (SEPs) for GFP‐Turbo‐HDEL and 2C1‐GFP‐Turbo, respectively (Figure 2C), using filtering criteria of false discovery rate (FDR) ≤ 0.05 and the cut‐off for the level of difference in the actual measurement (S_0_) = 1.0 by Perseus. Of those SEPs, ER luminal‐binding proteins (NbD042545.1; NbD049851.1; NbD015548.1) were significantly enriched in GFP‐Turbo‐HDEL samples, while ER membrane protein complex subunit‐like protein (NbD049803.1) was preferentially detected in 2C1‐GFP‐Turbo samples (Data S1). These findings suggest that the PL approach using GFP‐Turbo‐HDEL and 2C1‐GFP‐Turbo successfully discriminates between ER luminal and membrane proteomes, and that the two ER‐localising TurboID fusions can be appropriate controls to determine the precise location of proximity proteins of ER‐associated proteins of interest.

### 2.3 Identification and Subcellular Localization Prediction of the MET‐Specific Proximal Proteome

**FIGURE 3:**
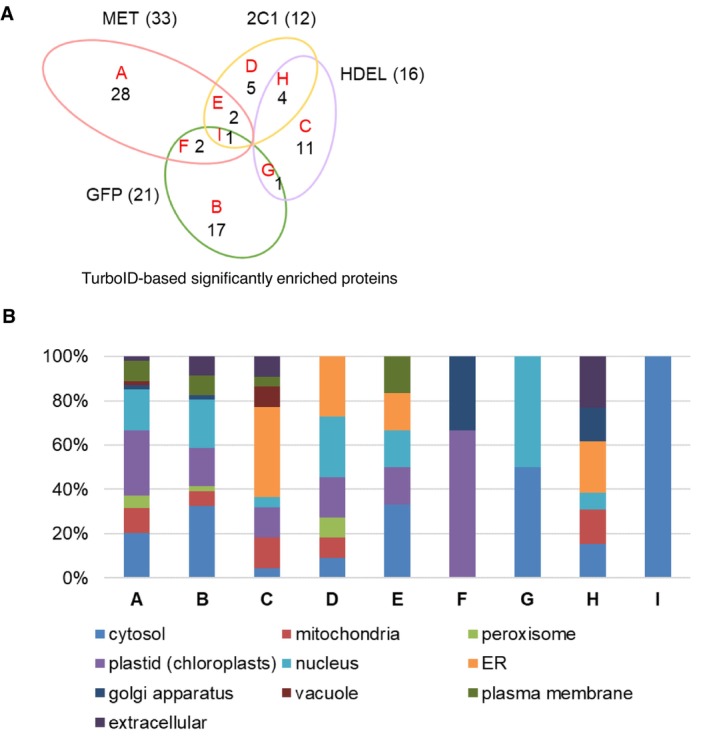
MET‐proximal proteome exhibits distinct subcellular localization patterns compared to cytosolic and endoplasmic reticulum (ER)‐localised controls. (A) Venn diagram of significantly enriched proteins (SEPs) identified by proximity labelling with four TurboID constructs. Groups represent proteins uniquely enriched by MET‐Turbo (Group A), GFP‐Turbo (Group B), GFP‐Turbo‐HDEL (Group C), or 2C1‐GFP‐Turbo (Group D), as well as the shared proteins between two constructs (Group E–H), or among three constructs (Group I). Numbers in parentheses indicate total SEPs identified for each construct. (B) Subcellular localization predictions and the localization proportion from each group using SUBA5, WoLF PSORT and ERPred algorithms. The detailed individual protein list is shown in Figure S2.

Under the same conditions as described above, we performed PL with MET‐Turbo alongside three controls: GFP‐Turbo, GFP‐Turbo‐HDEL and 2C1‐GFP‐Turbo. SEPs were defined through pairwise comparisons between all combinations using the filtering criteria of FDR ≤ 0.05 and S_0_ = 1.0, and subsequently classified into Groups A to I based on their enrichment patterns across the four baits (Figure 3A,B; Data S2). This systematic classification system allowed the categorization of SEPs as either unique or shared proteins across different bait‐specific proximal proteomes. A total of 33, 21, 16 and 12 SEPs were identified with MET‐Turbo, GFP‐Turbo, GFP‐Turbo‐HDEL and 2C1‐GFP‐Turbo, respectively (Figure 3A; Data S2). Of these, 28, 17, 11 and 5 proteins were exclusively enriched as unique interactors for each bait by MET‐Turbo, GFP‐Turbo, GFP‐Turbo‐HDEL and 2C1‐GFP‐Turbo (Groups A to D), respectively. Meanwhile, only a limited number of proteins (one to four) were identified as shared interactors between MET‐Turbo and 2C1‐GFP‐Turbo (Group E), MET‐Turbo and GFP‐Turbo (Group F), GFP‐Turbo and GFP‐Turbo‐HDEL (Group G), GFP‐Turbo‐HDEL and 2C1‐GFP‐Turbo (Group H) and MET‐Turbo, GFP‐Turbo and 2C1‐GFP‐Turbo (Group I). Interestingly, no enriched proteins were shared between MET‐Turbo and GFP‐Turbo‐HDEL, suggesting that MET and this luminal ER marker may not overlap in their subcellular localization sites. These results indicate that our PL‐based proximal proteomes provide the interactor information on TurboID‐fused baits localised separately around the ER.

To gain further insight into the spatial organisation of the MET‐Turbo proximal proteome, we predicted subcellular localization of all identified SEPs using SUBA5, WoLF PSORT and ERPred (Figure 3B; Figure S2). As expected, proteins exclusively enriched in GFP‐Turbo samples (Group B) were predominantly predicted to localise to the cytosol, nucleus, or other organelles, but not to the ER. Conversely, SEPs uniquely identified in GFP‐Turbo‐HDEL or 2C1‐GFP‐Turbo samples were largely predicted to localise to the ER, confirming the validity of our subcellular targeting approach (Figure 3B; Figure S2 Groups C and D).

Group C proteins, representing unique SEPs from GFP‐Turbo‐HDEL samples, include established ER luminal markers such as the HSP90‐like protein GRP94 (glucose‐regulated protein 94, NbD052671.1) and ER luminal‐binding proteins BiP and BiP1 (binding immunoglobulin protein, NbD042545.1 and NbD049851.1), which have been well characterised as ER lumen residents (Gething 1999; Marzec et al. 2012; Pobre et al. 2019). Group D, representing SEPs unique to 2C1‐GFP‐Turbo, contained the ER membrane complex subunit‐like protein EMC4 (NbD049803.1), confirming ER membrane localization specificity.

Surprisingly, the 28 proteins exclusively enriched in MET‐Turbo samples (Group A) and the five proteins identified as shared interactors for MET‐Turbo with GFP‐Turbo and/or 2C1‐GFP‐Turbo (Group E, F and I) were not predominantly predicted to localise to the ER. Instead, these proteins were largely predicted to associate with chloroplasts, nuclei, cytosol or mitochondria (Figure 3B; Figure S2). This unexpected localization pattern, combined with our observation that the MET domain forms distinct granules partly and closely associated with the ER network (Komatsu et al. 2021; Figure 1C), suggests that the MET domain may localise to specialised regions in close proximity to the ER membrane that interface with multiple organelles. The MET domain may partially associate with the cytosolic face of the ER membrane while simultaneously interacting with proteins from diverse subcellular compartments.

### 2.4 Functional Evaluation of MET‐Interacting Host Factors

To investigate the biological relevance of MET‐proximal proteins, we selected three candidates from the 28 Group A proteins for functional analysis. These candidates were strategically chosen to represent diverse cellular compartments and functional pathways associated with the early stages of viral infection. Specifically, we selected calcyclin‐binding protein (CACYBP, represented in this study as NbCBP), previously identified as an interactor of the ER‐resident ethylene response regulator CTR1 (Chien et al. 2024); remorin1.5 (NbREM1.5), a member of the plant‐specific plasma membrane‐associated remorin family involved in viral cell‐to‐cell movement (Ma et al. 2022); and calcium‐sensing receptor (NbCAS), which functions in salicylic acid (SA)‐mediated plant immunity (Li et al. 2022) and is potentially targeted by viral RNA silencing suppressors (Medina‐Puche et al. 2020; Zvereva et al. 2024). This selection allows for a broad assessment of how the MET interactome encompasses multiple defence and physiological pathways.

**FIGURE 4:**
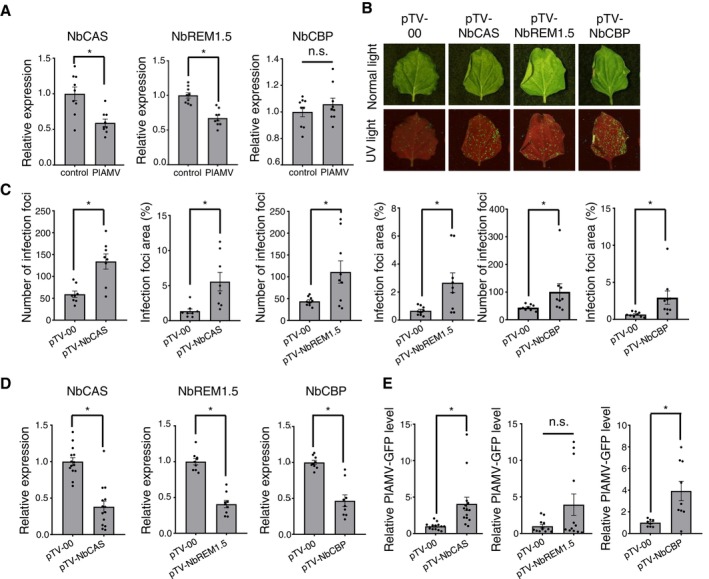
Functional characterisation of MET domain‐proximal host factors reveals their antiviral roles in Plantago asiatica mosaic virus (PlAMV) infection. (A) Relative expression levels of *NbCAS*, *NbREM1.5* and *NbCBP* in *Nicotiana benthamiana* leaves at 3 days post‐infiltration (dpi) with an infectious clone of PlAMV or an empty vector pCAMBIA1301 (control), determined by reverse transcription‐quantitative PCR (RT‐qPCR), with *NbPP2A* as a reference. Data represent means ± standard error of the mean (SEM) from three independent experiments. *p < 0.05, Student's *t* test. (B) Representative images of *N. benthamiana* leaves inoculated with PlAMV‐GFP virions under visible light (upper panels) and UV light (lower panels) at 3 dpi. Plants were pre‐inoculated with pTV‐00 (empty vector control), pTV‐NbCAS, pTV‐NbREM1.5, or pTV‐NbCBP for knockdown of each gene. (C) Numbers of the infection foci and the percentage of the total fluorescent area per inoculated leaf calculated from PlAMV‐GFP inoculated leaves. Data represent means ± SEM from three independent experiments with a total of 8 biological replicates of pTV‐NbCAS, 9 biological replicates of pTV‐NbREM1.5 and 9 biological replicates of pTV‐NbCBP‐inoculated plants. *p < 0.05, Student's *t* test. (D, E) Relative accumulation of *NbCAS*, *NbREM1.5* and *NbCBP* genes (D) and PlAMV‐GFP RNA (E) in inoculated leaves of pTV‐00 control and knockdown plants, quantified by RT‐qPCR with *NbPP2A* as a reference. Data represent means ± SEM from three independent experiments with a total of 14 biological replicates of pTV‐NbCAS, 12 biological replicates of pTV‐NbREM1.5 and 9 biological replicates of pTV‐NbCBP‐inoculated plants. *p < 0.05, Student's *t* test.

We first examined whether PlAMV infection affects the expression of these genes in *N. benthamiana*. Reverse transcription‐qPCR (RT‐qPCR) of total RNA extracted at 3 days post‐inoculation (dpi) revealed significant downregulation of *NbCAS* and *NbREM1.5* transcripts in PlAMV‐infected plants compared to mock‐inoculated plants, while *NbCBP* expression remained unchanged (Figure 4A). To investigate whether these host proteins influence PlAMV infection, we knocked down the expression of these genes using virus‐induced gene silencing (VIGS) with a tobacco rattle virus (TRV) vector. Plants were inoculated with TRV vectors carrying gene‐specific fragments (pTV‐NbCAS, pTV‐NbREM1.5, or pTV‐NbCBP; Table S1) or with empty TRV vector (pTV‐00) as control. At 10 dpi, PlAMV‐GFP virions were mechanically inoculated onto the upper uninoculated leaves. Three days after PlAMV‐GFP inoculation, we examined viral accumulation through fluorescence observation and RT‐qPCR. The number of GFP fluorescent foci and the percentage of the total fluorescent area per inoculated leaf increased significantly in all three knockdown plants compared to controls (Figure 4B,C), with significant reductions in target gene expression confirmed by RT‐qPCR (Figure 4D). Consistent with these observations, RT‐qPCR revealed significantly higher PlAMV‐GFP accumulation in *NbCAS*‐ and *NbCBP*‐knockdown plants compared to controls. *NbREM1.5*‐knockdown plants showed an increasing trend, although not statistically significant (Figure 4E). These results indicate that all three MET‐proximal factors function as antiviral components during PlAMV infection.

### 2.5 Interaction With PlAMV MET Causes the Subcellular Relocalization of MET‐Proximal Host Factors

**FIGURE 5:**
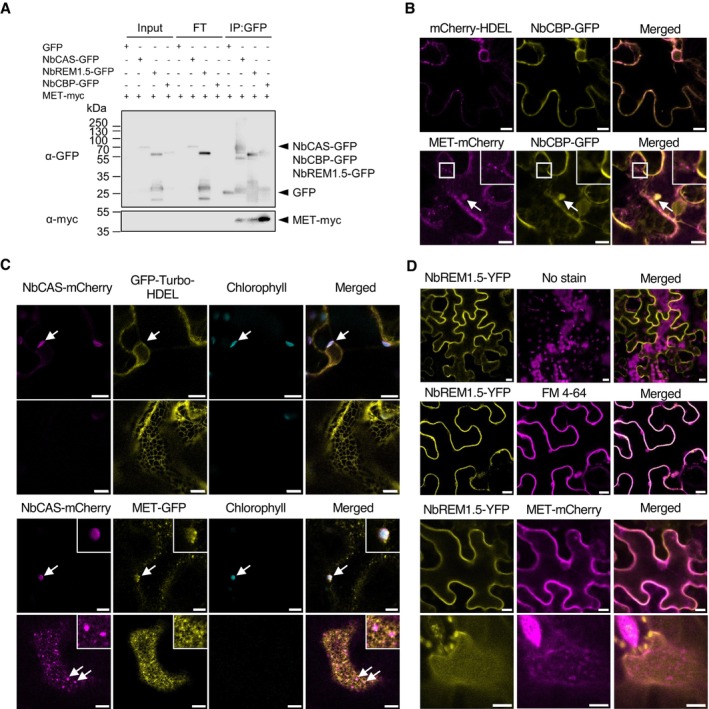
Association of MET domain and three selected MET‐proximal host factors, and subcellular relocalization of the three factors by co‐expression with MET. (A) Co‐immunoprecipitation assay. GFP‐fused selected host factors (NbCAS‐GFP, NbREM1.5‐GFP, or NbCBP‐GFP), myc‐fused MET domain (MET‐myc) and p19 silencing suppressor were co‐expressed in *Nicotiana benthamiana* leaves as indicated at the top. Total proteins were extracted at 3 days post‐infiltration (dpi) for co‐immunoprecipitation using GFP‐Trap agarose beads. Total protein extracts (Input), flow‐through (FT) samples and immunoprecipitates (IP:GFP) were analysed by immunoblotting using anti‐GFP and anti‐c‐Myc antibodies. Arrowheads represent expected band sizes. The target protein signals (MET‐myc) were below the detection limit in the Input and FT fractions but were successfully detected in the IP fractions due to enrichment by GFP‐Trap agarose beads. (B–D) Confocal microscopy analysis of subcellular colocalization between MET‐mCherry/GFP and selected host proteins in *N. benthamiana* epidermal cells at 1 dpi. mCherry‐HDEL and GFP‐Turbo‐HDEL were used as controls. (B) Co‐expression of MET‐mCherry and NbCBP‐GFP. White arrows indicate spherical bodies of MET‐mCherry colocalized with NbCBP‐GFP. The insets show the spot‐like small granules of MET‐mCherry observed at the endoplasmic reticulum (ER) network. (C) Co‐expression of NbCAS‐mCherry and MET‐GFP. Chlorophyll autofluorescence is shown in cyan. White arrows in the first panel indicate the chloroplast‐localization of NbCAS‐mCherry. White arrows in the third panel indicate the colocalization of MET‐GFP small granules with NbCAS‐mCherry at chloroplasts (shown in insets). White arrows in the fourth panel indicate the relocalization of NbCAS‐mCherry granules between the MET‐GFP granules (shown in insets). (D) The first and second rows represent the membrane‐localization pattern of NbREM1.5‐YFP, with (second row) or without (first row) FM4‐64 staining as a plasma membrane marker. The third and fourth rows represent a transverse section (third row) and a cellular surface view (fourth row) of cells co‐expressing NbREM1.5‐YFP and MET‐mCherry. Scale bars = 10 μm.

We next tested the interaction of the MET domain with each of the three proximal host factors. Co‐immunoprecipitation with anti‐GFP antibodies and subsequent immunoblotting with anti‐myc antibodies showed that the myc tag‐fused MET domain was co‐immunoprecipitated with all three GFP‐fused host factors, confirming the physical interaction between these factors and the MET domain (Figure 5A).

To examine the spatial relationship between these host factors and the MET domain, confocal microscopy analysis was performed. Co‐expression of MET‐mCherry with NbCBP‐GFP demonstrated their colocalization in larger spherical bodies (Figure 5B, bottom row), which moved together within the cytoplasmic area (Video S1). These large spherical bodies were absent when MET‐mCherry was not co‐expressed (Figure 5B, upper row). Notably, the spot‐like small granules of MET‐mCherry, which were observed at the ER network as previously reported (Komatsu et al. 2021), were associated with amorphously distributed NbCBP‐GFP (Figure 5B, insets in the bottom row). Co‐expression of NbCAS‐mCherry and GFP‐Turbo‐HDEL confirmed the chloroplast localization of NbCAS‐mCherry (Bai et al. 2022; Li et al. 2022) around the nucleus, but without any granule‐like fluorescence signals at the peripheral ER network (Figure 5C, first and second rows). In contrast, co‐expression of MET‐GFP and NbCAS‐mCherry revealed partial colocalization, with a subset of MET‐GFP small granules overlapping with NbCAS‐mCherry signals at chloroplast peripheries. Notably, NbCAS‐mCherry formed distinct granular structures in the cytoplasmic space between small granules of MET‐GFP, positioned in close proximity to the ER network (Figure 5C, insets in the third and fourth rows). This altered localization of NbCAS differs from previous reports describing its thylakoid‐localization (Bai et al. 2022; Li et al. 2022), suggesting potential relocalization of NbCAS by MET. Similarly, expression of NbREM1.5‐YFP with staining with a plasma membrane marker, FM4‐64, confirmed the uniform distribution of NbREM1.5‐YFP at the plasma membrane (Figure 5D, first and second rows). MET‐mCherry showed a shift in localization in the presence of NbREM1.5‐YFP, with granules becoming less prominent and redistributing closer to the plasma membrane (Figure 5D, third and fourth rows). This altered localization of MET‐mCherry contrasts with the pronounced granule formation of MET‐GFP observed when co‐expressed with mCherry‐HDEL (Figure 1B, fifth row), suggesting that NbREM1.5 might modulate the subcellular positioning of the MET domain. These colocalization and relocalization patterns suggest that the three MET‐proximal host factors may interact with PlAMV MET at specific subcellular sites.

## 3 Discussion

Comprehensive proteomic analysis of the ER remains technically challenging, while it is essential for elucidating mechanisms underlying (+)RNA virus replication, most of which occurs within ER‐associated environments with the help of various host factors. Conversely, plants may exploit this same cellular environment to deploy antiviral defence mechanisms. In this study, we established an ER‐associated proximity labelling approach with the appropriate ER‐localising controls (i.e., GFP‐Turbo‐HDEL and 2C1‐GFP‐Turbo). The subsequent liquid chromatography–tandem mass spectrometry (LC–MS/MS) and functional analyses identified host antiviral factors associated with the MET, a membrane‐associated replicase domain of PlAMV containing an amphipathic α‐helix that mediates membrane binding and virus replication (Komatsu et al. 2021).

Specific ER‐proximal labelling by our ER‐localising TurboID‐fused marker proteins was validated by the significant enrichment of ER luminal‐binding proteins (NbD042545.1, NbD049851.1 and NbD015548.1) for the ER lumen and ER membrane protein complex subunit‐like protein (NbD049803.1) for the ER membrane. This study shows that our ER‐associated marker control datasets can serve as the foundation for analysing the proximal proteome of the replicase from RNA viruses including PlAMV.

**FIGURE 6:**
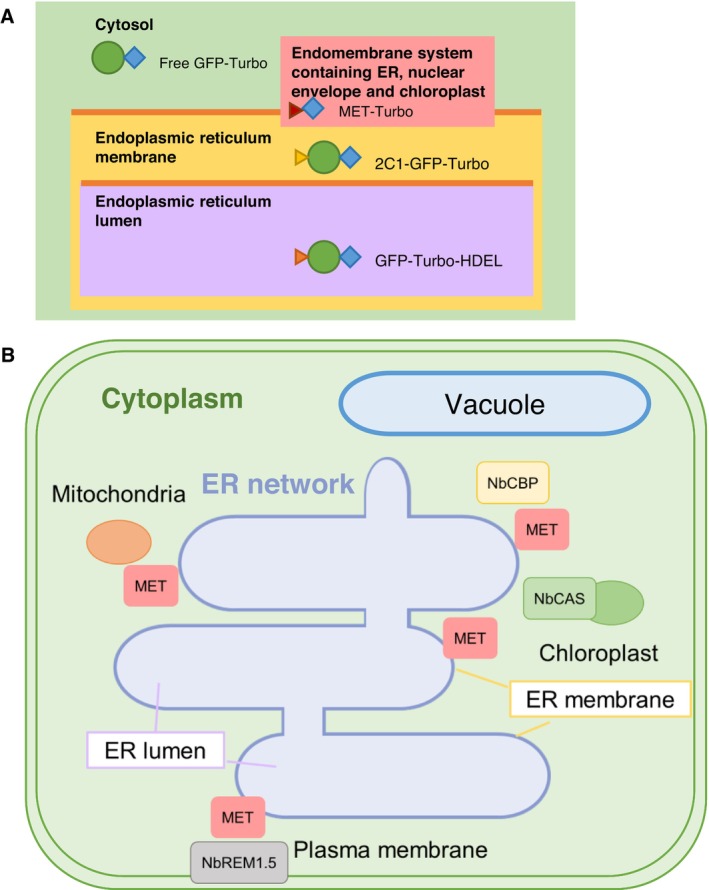
A model for the localization of MET domain of Plantago asiatica mosaic virus replicase and its interaction with host factors. (A) A relative distribution tendency of the TurboID‐fused MET domain (MET‐Turbo) compared with three controls: GFP‐Turbo, GFP‐Turbo‐HDEL and 2C1‐GFP‐Turbo. (B) Schematic diagram of the MET domain localization hypothesis. MET is likely to be located near the cytosol‐facing site of the endoplasmic reticulum (ER) membrane and possesses a unique proxiome.

Using these controls, we identified specific proximal proteins of MET‐Turbo based on comparison of its proxiome profile with those of GFP‐Turbo‐HDEL and 2C1‐GFP‐Turbo as well as GFP‐Turbo. The predicted subcellular localization of SEPs from MET‐Turbo further highlights the unique association of MET with the ER membrane, which differs from the ER‐localising controls, as summarised in our model illustration (Figure 6A). MET may localise to a specialised ER subdomain that connects with multiple organelles, including chloroplasts and mitochondria (Figure 6B), possibly via membrane contact sites (MCSs) as discussed below. The possible specialised localization of MET was corroborated by confocal microscopic observation of the partial colocalization of MET‐GFP‐Turbo with the ER network, which suggests that PlAMV exploits the ER network as a VRC formation site while simultaneously recruiting proteins from other subcellular organelles.

Recent studies have emphasised the critical importance of MCSs for inter‐organellar connection within plant cells, particularly those with the ER network (Mathur et al. 2022; Wang et al. 2023). MCSs are emerging as pivotal subcellular hubs that organise both viral replication and plant defence responses. Strikingly, the VAP27 protein, which mediates the formation of MCSs between ER and small organelles (Renna et al. 2024; Wang et al. 2023), co‐localises with potyviral 6K2 protein, facilitating the fusion of ER‐derived viral replication vesicles with chloroplasts to support efficient viral replication (Wei et al. 2013). Although our MET‐proximal dataset does not include VAP27, this previous finding suggests a direct link between MCSs and the viral infection process. To fully understand how viruses exploit MCSs, advanced techniques are needed to investigate MCS contents and unravel the molecular mechanisms operating within this special microenvironment. Recently reported techniques employing TurboID‐fused tethering proteins for ER tubule‐actin complexes could elucidate protein networks at ER–cytoskeleton interfaces (Merta et al. 2025; Zang et al. 2021). Moreover, a split‐BioID method called Contact‐ID, which has been successfully applied to study ER–mitochondria contact sites in live mammalian cells, represents a promising tool for investigating MCS‐specific proxiomes in plants (Kwak et al. 2020). In the future, integration of these methods with ER‐targeted proximity labelling could illuminate the specific ER subdomains responsible for their functions in the inter‐organellar MCSs.

Several well‐documented examples illustrate how viruses have evolved to exploit MCSs as strategic platforms for replication. Previous studies have shown that tomato bushy stunt virus recruits glycolytic enzymes into its VRC to generate local ATP production for efficient replication (Chuang et al. 2017; Molho et al. 2021). Recent potexvirus research highlights the relationship between the glycolytic metabolon and the replication process of bamboo mosaic virus (BaMV), which occurs not only at the ER network but also at its MCSs with chloroplasts and mitochondria. The chloroplastic phosphoglycerate kinase (chPGK) assists in targeting BaMV RNA to chloroplasts during the early replication (Cheng et al. 2013), while genomic and subgenomic RNAs are subsequently released from chloroplasts via ATG8‐mediation (Huang et al. 2019). Given that glycolytic metabolons connect chloroplasts and mitochondria (Zhang et al. 2020), current models of BaMV replication suggest that VRCs may recruit glycolytic pathway members to form large aggregates involving mitochondria surrounded by the ER (Lin et al. 2025). MET‐proximal host factors identified in this study include chloroplast and mitochondrial proteins involved in ATP generation and the glycolytic pathway, such as ATP synthases and PGK (Figure 3B; Figure S2). Although future studies are needed to experimentally confirm the proviral roles of these proteins, this proxiome composition suggests that the MET‐proxiome reflects a critical aspect of VRC functions: recruiting host factors from small organelles to create an ATP‐enriched microenvironment.

Plants do not remain passive to viral infection. Rather, they actively employ antiviral defences through multiple mechanisms. Recent research has revealed that antiviral RNA silencing protein Argonaute 2 interacts with a chloroplast‐localised RNA helicase 3 at ER‐chloroplasts MCSs to facilitate small RNA loading (Huang et al. 2025), providing a spatial foundation for antiviral RNA silencing. Importantly, MCSs participate in modulating inter‐organellar calcium dynamics, which are essential for plant immunity, including the priming of RNA silencing (Wang et al. 2021) and SA‐mediated resistance (Du et al. 2009). This connection between MCSs and calcium signalling is further supported by our findings on NbCAS (calcium‐sensing receptor) as a MET‐proximal antiviral factor against PlAMV infection. NbCAS relocates proximal to the ER network upon co‐expression with the MET domain (Figure 5C), which may contribute to the activation of SA‐related defence responses (Medina‐Puche et al. 2020) (Figure 4). These observations strengthen the hypothesis that calcium signalling, modulated through MCSs, influences viral infection through direct interactions with viral factors and subsequent subcellular relocalization.

The importance of host factor relocalization during virus infection is well‐documented also in the studies of remorin protein family (REM), which exhibits both proviral and antiviral functions. Research on potyviruses demonstrates that interactions with viral VPg trigger the relocalization of sugarcane REM1.5 and *Arabidopsis* REM1.2/1.3 from the plasma membrane to the cytosol, facilitating virus infection (Cheng et al. 2020; Yang et al. 2024). Similarly, NtREM1.2 relocalization from the uniform plasma membrane to form aggregates has been observed during the infection by tomato mosaic virus and potato virus X (Chen et al. 2025; Sasaki et al. 2018). Conversely, studies of tobacco mosaic virus (TMV) reveal that palmitoylation‐dependent plasma membrane localization is essential for the ability of NbREM1.5 to restrict TMV cell‐to‐cell movement (Ma et al. 2022). In our study, co‐expression of NbREM1.5, which is an antiviral host protein against PlAMV (Figure 4), with the MET domain resulted in partial relocalization of the MET domain closer to the plasma membrane raft (Figure 5D). We cannot exclude the possibility that the relocalization of the MET domain, whose correct localization would be important for PlAMV replication, away from the strict ER association by NbREM1.5, is responsible for the restriction of PlAMV infection by NbREM1.5. Future studies are needed to investigate the mechanisms underlying this mutual relocalization and elucidate how NbREM1.5 participates in antiviral responses targeting PlAMV replicase.

Another notable finding in this study is the identification of NbCBP as an antiviral protein. Calcyclin binding protein (CacyBP; CBP) is a multifunctional protein that participates in calcium‐dependent ubiquitination and proteasomal degradation (Filipek 2006; Matsuzawa and Reed 2001) and serves as an Hsp90 chaperone during stress responses in animal systems (Góral et al. 2016). Although the role of CBP in plant–pathogen interactions remains poorly understood, findings in our current study led us to hypothesise that the colocalization of NbCBP with MET (Figure 5B) may trigger ubiquitination and subsequent degradation of MET. Furthermore, considering the recent evidence that CBP acts as a proximal interactor with the ER‐resident protein kinase, constitutive triple response 1 (CTR1) (Chien et al. 2024), as well as the identification of another kinase PGK as a MET‐proximal protein in this study (Figure 3B; Figure S2), it is possible that CBP may facilitate phosphorylation of MET by recruiting host protein kinases. Such phosphorylation could modulate MET function or subcellular localization to disturb PlAMV infection, as previously demonstrated regulatory roles of phosphorylation of a replicase in replication of many animal and plant (+)RNA viruses (Jakubiec and Jupin 2007).

Based on our results, we conclude that our methodology for ER‐related proximity labelling is effective. Additionally, the findings from our MET proxiome show promise for future mapping of the VRC proxiome under virus infection conditions. Considering the previous report that the ER luminal proteins are responsible for brome mosaic virus genomic RNA replication, it is possible that ER luminal proteins are also involved in PlAMV replication (Nishikiori and Ahlquist 2018). In our current study, we used MET domain transient expression but not the whole virus infection condition, which may explain why our MET‐proxiome results show stronger association with the cytosol‐facing ER membrane compartment (Figure 6A) and highlights the need to further investigate the replicase‐proxiome under conditions that more closely reflect viral infection. To investigate the replicase‐proxiome in a virus infection context, we propose adapting our recently developed antibody‐probe system (Ishihara et al. 2025). Building upon previous studies that have leveraged antigen–antibody interactions to enhance the specificity of TurboID labelling (Lee et al. 2023; Xiong et al. 2021), we hypothesise that substituting the fluorescent protein with TurboID would enable spatiotemporally controlled expression of proximity labelling components specifically during active viral replication.

## 4 Experimental Procedures

### 4.1 Plants, Virus, Agroinfiltration and Virion Inoculation

*Nicotiana benthamiana* plants were grown at 25°C under 16 h light/8 h dark cycles. For TurboID‐fused protein analysis, 4‐week‐old plants were agroinfiltrated with *Agrobacterium tumefaciens* (OD_600_ = 1.0) harbouring each TurboID‐fusion construct. For virus inoculation using agroinfiltration, pLi1, an infectious clone of the PlAMV Li1 isolate (Ozeki et al. 2006), was used with OD₆₀₀ = 1.0. *Agrobacterium* carrying pCAMBIA1301, which expresses the β‐glucuronidase (*GUS*) gene, served as a control. For virus‐induced gene silencing (VIGS), 3‐week‐old *N. benthamiana* plants were infiltrated with a mixture of *Agrobacterium* cultures (OD₆₀₀ = 0.1 each) containing pBINTRA6 and either pTV‐00 (empty vector) (Shi et al. 2021), pTV‐NbCAS, pTV‐NbREM1.5, or pTV‐NbCBP. Purified virions of PlAMV expressing GFP PlAMV (PlAMV‐GFP) were mechanically inoculated using carborundum as described previously (Matsuo et al. 2019; Minato et al. 2014).

### 4.2 Plasmid Construction

Plasmids for proximity labelling were constructed based on a binary vector pCAMBIA1301.1‐sGFP (Komatsu et al. 2021). GFP and GFP‐HDEL fragments were PCR‐amplified using the plasmids pCAMBIA1301.1‐GFP and pCAMBIA‐1301‐1‐ER‐GFP5‐HA‐HDEL as templates (Ishihara et al. 2025), respectively, with primer sets pCAMBIA25‐F/GFP‐SpeI‐Turbo‐R and pC1301‐1‐SalI‐ER‐GFP5′‐F/pC1301‐1‐ERGFP5‐SpeI‐Turbo‐R. The MET domain was amplified from pLi1 using primers pCAMBIA‐MET‐F/MET‐SpeI‐Turbo‐R. TurboID fragments were amplified from pENTR_L1‐YFP‐Turbo‐L2 (Addgene plasmid #127350) using MET‐SpeI‐Turbo‐F/Turbo‐myc‐pCAMBIA‐R for MET‐Turbo and pC1301‐1‐GFP5‐SpeI‐Turbo‐F/pCAM‐GFP‐Turbo2‐myc‐HDEL‐R for GFP‐Turbo‐HDEL. These TurboID fragments were combined with their respective partner fragments (MET or GFP‐HDEL) by overlap extension PCR using primer sets pCAMBIA‐MET‐F/Turbo‐myc‐pCAMBIA‐R or pC1301‐1‐SalI‐ER‐GFP5′‐F/Turbo‐myc‐HDEL‐pCAM‐R2 to generate MET‐Turbo and GFP‐Turbo‐HDEL. The resulting fusion fragments were inserted into SalI/BamHI‐digested pCAMBIA1301.1‐GFP using In‐Fusion Snap Assembly Master Mix (Takara Bio) to generate pCAMBIA‐MET‐Turbo and pCAMBIA‐ER‐GFP‐Turbo. GFP was inserted into NcoI/BamHI‐digested pCAMBIA‐MET‐Turbo to produce pCAMBIA‐GFP‐Turbo. For the 2C1 construct, a synthetic DNA fragment encoding 27 amino acids (Addgene plasmid #79055; Lam et al. 2014) (Table S2) of cytochrome P450 2C1 (Ahn et al. 1993) was obtained (Eurofins Genomics) and fused with a GFP‐Turbo fragment amplified from pCAMBIA‐GFP‐Turbo using 2C1‐31aa‐GFP‐F/Turbo‐Myc‐pCAMBIA‐R. Both fragments were inserted into NcoI/BamHI‐digested pCAMBIA1301.1‐GFP to create pCAMBIA‐2C1‐GFP‐Turbo. To construct pCAMBIA‐MET‐GFP‐Turbo, the GFP‐Turbo fragment was amplified using pCAM‐MET‐GFP‐F/pCAM‐MET‐GFP‐Turbo‐myc‐R using pCAMBIA‐GFP‐Turbo as a template and inserted into SpeI/BamHI‐digested pCAMBIA‐MET‐Turbo.

To construct plasmids for transient overexpression of host factors, cDNA fragments of *NbCAS* and *NbCBP* (Table S3) were amplified by reverse transcription PCR (RT‐PCR) with *N. benthamiana* total RNA as a template, with primer sets pCAMBIA‐SalI‐NbCAS‐F/pCAM‐NbCAS‐SpeI‐R‐new and pCAM‐calcyclin‐F/calcyclin‐pCAM‐R, respectively. RT‐PCR was performed using the PrimeScript II High Fidelity One Step RT‐PCR Kit (Takara Bio) according to the manufacturer's instructions. The resulting PCR products were cloned into SalI/SpeI‐digested pCAMBIA1301.1‐sGFP/mCherry (Komatsu et al. 2021) using In‐Fusion Snap Assembly Master Mix (Takara Bio) to generate pCAMBIA‐NbCAS‐sGFP, pCAMBIA‐NbCAS‐mCherry and pCAMBIA‐NbCBP‐sGFP. A cDNA fragment of *NbREM1.5* (Table S3) was amplified by RT‐PCR using the PrimeScript RT reagent Kit with gDNA Eraser (Takara Bio), with *N. benthamiana* total RNA as a template and with primer sets NbREM1.5/GW/F01 and NbREM1.5/GW/R01, followed by a second PCR with att adaptor primers (attB1 adaptor primer/attB2 adaptor primer) (Sasaki et al. 2009). The second PCR product was purified and introduced into pDONR/Zeo (Thermo Fisher Scientific). pART27‐35Sa‐NbREM1.5‐sGFP and pGreenII‐NbREM1.5‐YFP were constructed via LR recombination between the resulting entry clone (pDONR/Zeo‐NbREM1.5) and the destination vector, pART27‐35Sa‐GWB‐sGFP and pGreenII‐GWC‐YFP, which was generated via BP recombination of pDONR/Zeo with pGreenII‐MDP92‐YFP (Yoshikawa et al. 2024).

To construct plasmids for host factor knockdown, target sequences for *NbCAS*, *NbREM1.5* and *NbCBP* were designed using pssRNAit (https://www.zhaolab.org/pssRNAit/). Gene fragments were amplified by RT‐PCR from *N. benthamiana* total RNA using primer sets pTV‐SpeI‐NbCAS‐F/pTV‐NbCAS‐KpnI‐R, NbREM1.5‐pTV‐F/pTV‐NbREM1.5‐R and NbCBP‐pTV‐F/pTV‐NbCBP‐R, which included overlapping sequences for In‐Fusion cloning. The PCR products were inserted into SpeI/KpnI‐digested pTV‐00 using In‐Fusion Snap Assembly Master Mix (Takara Bio) to generate pTV‐NbCAS, pTV‐NbREM1.5 and pTV‐NbCBP.

All PCRs for insert fragment amplification were performed using KOD‐Plus‐Neo (Toyobo) according to the manufacturer's instructions. All constructed plasmids were confirmed by restriction enzyme digestion and Sanger sequencing (Eurofins Genomics, Tokyo, Japan). All primers used for plasmid construction are listed in Table S4.

### 4.3 Confocal Microscopy

Imaging analyses were performed using a confocal laser scanning microscope (AX/AX R, Nikon) as described previously (Ishihara et al. 2025). For plasma membrane visualisation, leaves were infiltrated with 50 μM BioTracker 640 Red C2 (FM4‐64) Synaptic Dye (Sigma‐Aldrich) approximately 5–15 min prior to imaging. GFP, YFP, mCherry, FM4‐64 and chloroplast autofluorescence were excited by lasers with wavelengths of 488, 488, 561, 488 and 640 nm, and detected at 509–551, 518–551, 571–625 663–738 and 662–737 nm wavelength ranges, respectively. All fluorescence images were acquired with sequential scanning, and image processing was performed with ImageJ/Fiji software (https://imagej.net/software/fiji).

### 4.4 Protein Fractionation and Biochemical Treatment

Fractionation and biochemical analyses were performed as described previously (Komatsu et al. 2021). Leaf tissue (0.3 g) agroinfiltrated with *Agrobacterium* containing TurboID‐fused constructs was harvested after 24 h post‐infiltration (hpi). Leaves were ground in liquid nitrogen, and total proteins were extracted by 0.5 mL buffer A (50 mM Tris–HCl pH 7.5, 120 mM KCl, 15 mM MgCl_2_, 20% glycerol, 0.1% v/v 3‐mercapto‐1,2‐propanediol, one tablet of cOmplete Mini protease inhibitor cocktail per 10 mL of buffer). After two centrifugation steps at 1000 *g* for 10 min at 4°C to remove debris, the supernatant was centrifuged at 30,000 *g* for 30 min at 4°C (himac CS100GXL, Hitachi). The resulting supernatant was collected as the S30 fraction, and the pellet was resuspended in 100 μL buffer A containing either 0.1 M Na_2_CO_3_ or 2% Triton X‐100 (TX‐100) and incubated on ice for 30 min, with standard buffer A serving as a control. After incubation, the solution was again centrifuged at 30,000 *g* for 30 min at 4°C, and the supernatant was collected as the S fraction, and the pellet was resuspended in 100 μL buffer A to obtain the P fraction.

### 4.5 Sample Preparation for Proximity Labelling and Mass Spectrometry

At 21 h after agroinfiltration with TurboID‐fused constructs (OD_600_ = 1.0), infiltrated leaves were treated with biotin solution (50 μM biotin in 10 mM MgCl_2_). At 3 h after the biotin infiltration, leaf tissue (0.7 g) was harvested and quickly stored at −80°C until protein extraction. Stored leaf samples were ground in liquid nitrogen. Total proteins were extracted with 1 mL RIPA buffer (50 mM Tris–HCl pH 7.5, 500 mM NaCl, 1 mM EDTA, 1% v/v NP40, 0.1% w/v sodium dodecyl sulphate (SDS), 0.5% w/v sodium deoxycholate, 1 mM dithiothreitol [DTT], one tablet of cOmplete Mini protease inhibitor cocktail [Roche] per 10 mL of buffer) followed by centrifugation at 16,500 *g* for 10 min at 4°C to remove debris. A 20 μL aliquot of the supernatant was collected for immunoblot analysis to confirm biotinylation by TurboID. The remaining supernatant underwent methanol/chloroform precipitation (Melkonian et al. 2022) to remove excess free biotin. The resulting pellet was resuspended with 500 μL SDT buffer (100 mM Tris–HCl pH 7.5, 4% SDS, 0.1 M DTT) and incubated with shaking at 200 rpm for 30 min at 22°C.

The SDT buffer‐treated sample was diluted with seven volumes of Tris‐buffered saline (TBS; 50 mM Tris–HCl, 150 mM NaCl, pH 7.5). To enrich biotinylated proteins, 100 μL of streptavidin‐coated magnetic beads (Streptavidin Mag Sepharose, Cytiva), prewashed with TBS, were added to the sample. After overnight incubation at room temperature, the beads were collected by centrifugation at 1500 *g* for 10 min and washed three times with TBS containing 2 M urea.

Biotinylated proteins enriched with streptavidin beads were processed into peptides via on‐bead trypsin digestion. Washed beads were treated with 25 μL treatment buffer 1 (50 mM Tris–HCl pH 7.5, 2 M urea, 1 mM DTT, 5 μg/mL trypsin) and incubated with shaking at 200 rpm for 40 min at 30°C. The digested solution was transferred to a new tube, and the beads were further treated with 50 μL treatment buffer 2 (50 mM Tris–HCl pH 7.5, 2 M urea, 5 μM iodoacetamide). This solution was combined with the initial digest and incubated overnight at 32°C with shaking at 500 rpm. Following overnight incubation, the solution was treated with 5 μL of 10% trifluoroacetic acid (TFA) for trypsin inactivation before LC–MS/MS analysis.

### 4.6 Mass Spectrometry and Subcellular Localization Prediction

The on‐bead digested samples were analysed using the LTQ‐Orbitrap XL mass spectrometer (Thermo Fisher Scientific). The mass spectra were searched against the NbDE2019BMCgenomics database (Kourelis et al. 2019) using MASCOT Server v. 2.4.1.

The raw data were analysed against the NbDE2019BMCgenomics database using MaxQuant v. 2.2.0.0. The proteome files were complemented with reverse decoy sequences and common contaminants by MaxQuant. Carbamidomethyl cysteine was set as a fixed modification, while methionine oxidation and protein N‐terminal acetylation were set as variable modifications. Digestion parameters were set to ‘Trypsin/P’.

Statistical analyses of the three biological replicates of the MaxQuant analysed proteomics data were performed using Perseus v. 2.0.7.0. Measured values were log_2_‐transformed, and only proteins with two or more valid values were retained for subsequent analysis. Missing values were imputed using Perseus default settings. Statistical analyses to identify enriched interacting proteins were carried out using two‐sample *t* tests coupled with permutation‐based false discovery rate (FDR) correction and the cut‐off for the level of difference in the actual measurement (S_0_). Proteins were classified as SEPs if they had an FDR ≤ 0.05 and S_0_ = 1, and a volcano plot was generated under these conditions.

The SEPs were initially annotated with descriptions containing predicted GenBank numbers. These GenBank numbers were queried in NCBI, and the resulting nucleotide sequences were further analysed by BLAST with the NbenBase database (https://nbenthamiana.jp/) to define gene names and descriptions based on orthologs in *Arabidopsis thaliana* . For subcellular localization prediction, the protein sequences of the SEPs were analysed using SUBA5 (https://suba.live/) based on its Predictions section, and the predicted result was double‐checked by the WoLF PSORT (https://www.genscript.com/wolf‐psort.html). The ER‐localised characteristic of each protein was double‐checked by the ERPred (http://proteininformatics.org/mkumar/erpred/).

### 4.7 Co‐Immunoprecipitation

*Agrobacterium* expressing MET‐myc was co‐infiltrated with NbCAS‐GFP, NbREM1.5‐GFP, or NbCBP‐GFP into 3‐ to 4‐week‐old *N. benthamiana* leaves. Approximately 0.3 g of agroinfiltrated leaves were collected 72 hpi and applied for the total protein extraction and immunoprecipitation by the GFP‐Trap agarose beads (ChromoTek) following our previous study (Shinji et al. 2023).

### 4.8 Immunoblot Analysis

Protein extracts from co‐immunoprecipitation, chemical treatment, or proximity labelling experiment were mixed with equal volumes of 2 × SDS sample buffer (Bio‐Rad) containing 5% v/v 3‐mercapto‐1,2‐propanediol and incubated for 5 min at 95°C. Samples were separated on 12.5% SDS‐PAGE gels (ATTO) and transferred to polyvinylidene difluoride (PVDF) membranes (Merck Millipore) using the wet transfer method at 500 mA, 100 V for 1 h. Membranes were blocked with Blocking One (NACALAI TESQUE) for 1 h at room temperature, washed with phosphate‐buffered saline containing 1% Tween‐20 (PBS‐T), and then incubated with antibody solutions prepared in Can Get Signal Immunoreaction Enhancer Solution (Toyobo). For detection of myc‐tagged proteins, mouse anti‐Myc tag antibody (Merck Millipore, clone 4A6; 1:1000 dilution) and horseradish peroxidase (HRP)‐conjugated goat anti‐mouse IgG (32430) (Thermo Fisher Scientific, #32430; 1:2000) were used. Protein biotinylation was detected using streptavidin‐HRP (Abcam, #ab7403; 1:4000) with the iBind Solution kit (Thermo Fisher Scientific) according to the manufacturer's protocol. Signals were detected using ImmunoStar LD substrates (Fujifilm Wako Pure Chemical Co.), and images were captured using LAS‐3000 (Fujifilm).

### 4.9 RNA Extraction and RT‐qPCR

Total RNA extraction from plants, cDNA synthesis, and qPCR were performed essentially as described previously (Komatsu et al. 2021). Total RNA was extracted using ISOGEN (Nippon Gene) according to the manufacturer's instructions. 2 μg of total RNA was treated with RQ1 RNase‐Free DNase (Promega), and the DNase‐treated RNA was used as template for cDNA synthesis with ReverTra Ace (Toyobo) according to the manufacturer's protocol.

Virus accumulation and host gene expression were relatively quantified by normalisation to the reference gene *NbPP2A* using a primer set NbPP2A‐F and NbPP2A‐R (Zhang et al. 2019). Primer sets qPCR‐NbCAS‐1F/qPCR‐NbCAS‐1R, qPCR‐NbREM1.5‐3F/qPCR‐NbREM1.5‐3R and qPCR‐NbCBP‐2F/qPCR‐NbCBP‐2R, designed based on Niben101Scf18639g00026.1, Niben101Scf08587g00002.1 and Niben101Scf03094g05009.1, were used for the quantification of the expression of *NbCAS*, *NbREM1.5* and *NbCBP* genes, respectively. A primer set PlAMV3877F/PlAMV4010R was used to quantify the accumulation of PlAMV‐GFP (Tanaka et al. 2019). GoTaq qPCR Master Mix (Promega) was used for qPCR, and the primer sequences are shown in the Table S4.

### 4.10 Fluorescence Observation of Virus Infection

Fluorescent infection spots of PlAMV‐GFP in host factor‐knockdown *N. benthamiana* plants by VIGS were visualised with a hand‐held UV lamp (UVGL‐58; Funakoshi) in a darkroom and photographed with a digital camera EOS90D (Canon).

## Author Contributions

**Yi‐Chen Chang:** writing – original draft, writing – review and editing, investigation, validation, data curation, formal analysis, visualization. **Sae Takatsuka:** investigation, methodology, formal analysis. **Aki Eguchi:** investigation. **Nobumitsu Sasaki:** methodology, visualization, investigation, writing – review and editing. **Yoshiyuki Itoh:** methodology, investigation. **Miki Hisada:** methodology, investigation. **Tsutomu Arie:** supervision, resources. **Ken Komatsu:** conceptualization, writing – review and editing, supervision, funding acquisition, project administration.

## Funding

This work was supported by the Japan Society for the Promotion of Science (23H02211).

## Conflicts of Interest

The authors declare no conflicts of interest.

## Supporting information


**Figure S1:** The immunoblotting data of the protein accumulation and biotinylation efficiency of the Turbo‐fusion proteins corresponding to Figure 1.

**Figure S2:** Subcellular localization predictions for individual proteins from each group corresponding to Figure 3B.

**Video S1:** Time‐lapse confocal microscopy of NbCBP‐GFP and MET‐mCherry co‐expression in the *Nicotiana benthamiana* epidermal cells. Imaging was performed with a frame interval of 20 s for each channel over a total period of 3.3 min (10 frames per channel). Scale bar = 10 μm.

**Data S1:** The SEPs enriched in GFP‐Turbo‐HDEL or 2C1‐GFP‐Turbo, comparison between the overlap proteins of HDEL‐ and 2C1‐captured proteins and total protein lists of HDEL‐ and 2C1‐captured proteins.

**Data S2:** Total SEPs of MET‐Turbo, GFP‐Turbo‐HDEL, 2C1‐GFP‐Turbo, or GFP‐Turbo identified with the pairwise comparison of each two baits.

**Table S1:** The target gene fragments for construction of host factors knockdown.

**Table S2:** The synthetic DNA fragment encoding 27 amino acids of cytochrome P450 2C1.

**Table S3:** cDNA sequence used for construction of transient overexpression of host factors.

**Table S4:** Primers used in this study.

## Data Availability

The data that support the findings of this study are available from the corresponding author upon reasonable request.
