# Supplementary material for: Proximity Labelling‐Based Proteomics Identifies Antiviral Host Factors Associated With the Potexvirus Replicase
Source: Mol Plant Pathol. 2026 Mar 19;27(3):e70239. doi: 10.1111/mpp.70239 (PMC13097338; doi:10.1111/mpp.70239)
Supplement: Supplementary file 1 — Figure S1: The immunoblotting data of the protein accumulation and biotinylation efficiency of the Turbo‐fusion proteins corresponding to Figure 1. [file MPP-27-e70239-s002.pptx]

## Slide 1
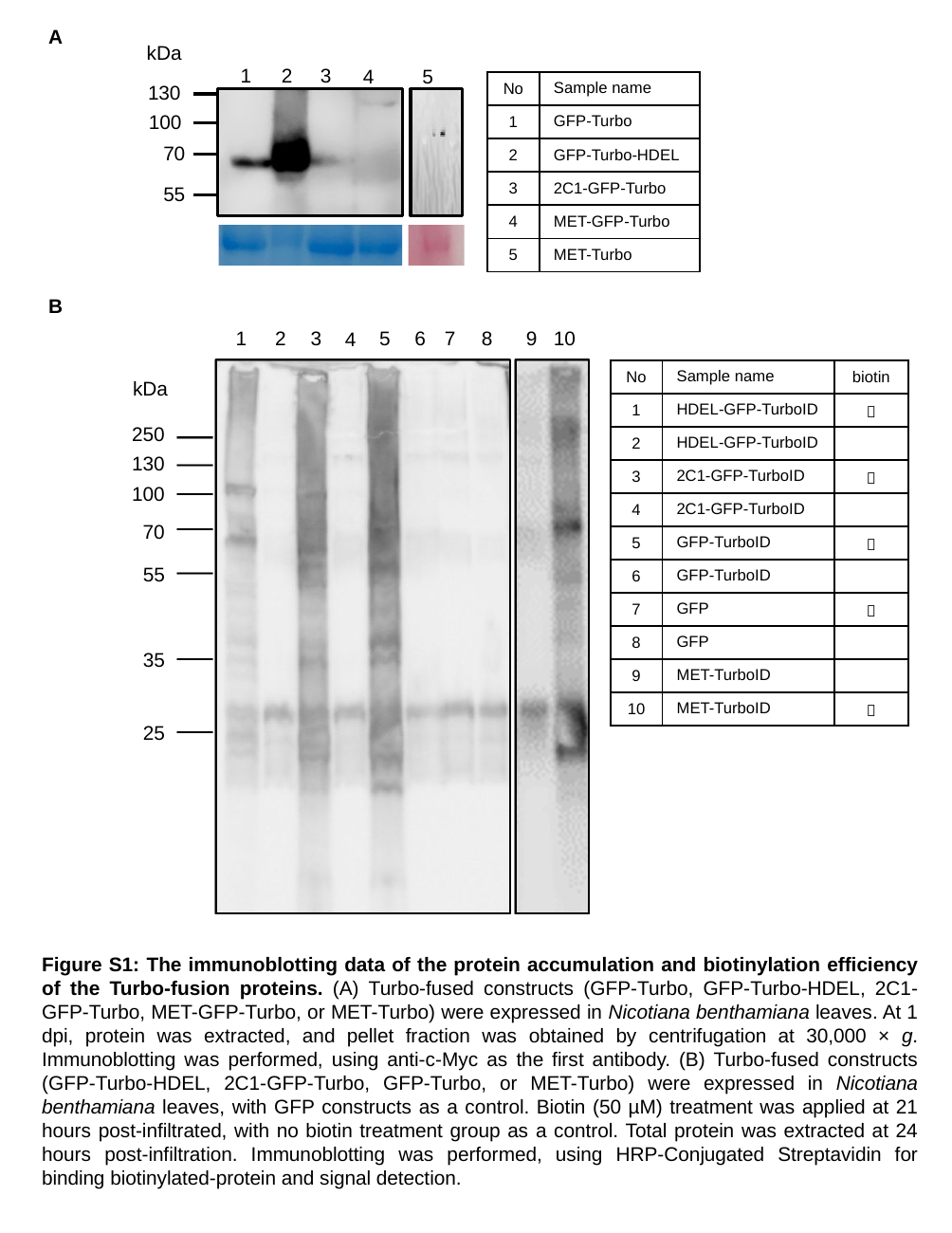

A
kDa
1
2
3
5
4
| No | Sample name |
| --- | --- |
| 1 | GFP-Turbo |
| 2 | GFP-Turbo-HDEL |
| 3 | 2C1-GFP-Turbo |
| 4 | MET-GFP-Turbo |
| 5 | MET-Turbo |
130
100
70
55
B
6
7
8
1
2
3
10
5
9
4
| No | Sample name | biotin |
| --- | --- | --- |
| 1 | HDEL-GFP-TurboID |  |
| 2 | HDEL-GFP-TurboID | |
| 3 | 2C1-GFP-TurboID |  |
| 4 | 2C1-GFP-TurboID | |
| 5 | GFP-TurboID |  |
| 6 | GFP-TurboID | |
| 7 | GFP |  |
| 8 | GFP | |
| 9 | MET-TurboID | |
| 10 | MET-TurboID |  |
kDa
250
130
100
70
55
35
25
Figure S1: The immunoblotting data of the protein accumulation and biotinylation efficiency of the Turbo-fusion proteins. (A) Turbo-fused constructs (GFP-Turbo, GFP-Turbo-HDEL, 2C1-GFP-Turbo, MET-GFP-Turbo, or MET-Turbo) were expressed in Nicotiana benthamiana leaves. At 1 dpi, protein was extracted, and pellet fraction was obtained by centrifugation at 30,000 × g. Immunoblotting was performed, using anti-c-Myc as the first antibody. (B) Turbo-fused constructs (GFP-Turbo-HDEL, 2C1-GFP-Turbo, GFP-Turbo, or MET-Turbo) were expressed in Nicotiana benthamiana leaves, with GFP constructs as a control. Biotin (50 µM) treatment was applied at 21 hours post-infiltrated, with no biotin treatment group as a control. Total protein was extracted at 24 hours post-infiltration. Immunoblotting was performed, using HRP-Conjugated Streptavidin for binding biotinylated-protein and signal detection.
