# Supplementary material for: Proximity Labelling‐Based Proteomics Identifies Antiviral Host Factors Associated With the Potexvirus Replicase
Source: Mol Plant Pathol. 2026 Mar 19;27(3):e70239. doi: 10.1111/mpp.70239 (PMC13097338; doi:10.1111/mpp.70239)
Supplement: Supplementary file 2 — Figure S2: Subcellular localization predictions for individual proteins from each group corresponding to Figure 3B. [file MPP-27-e70239-s007.pptx]

## Slide 1
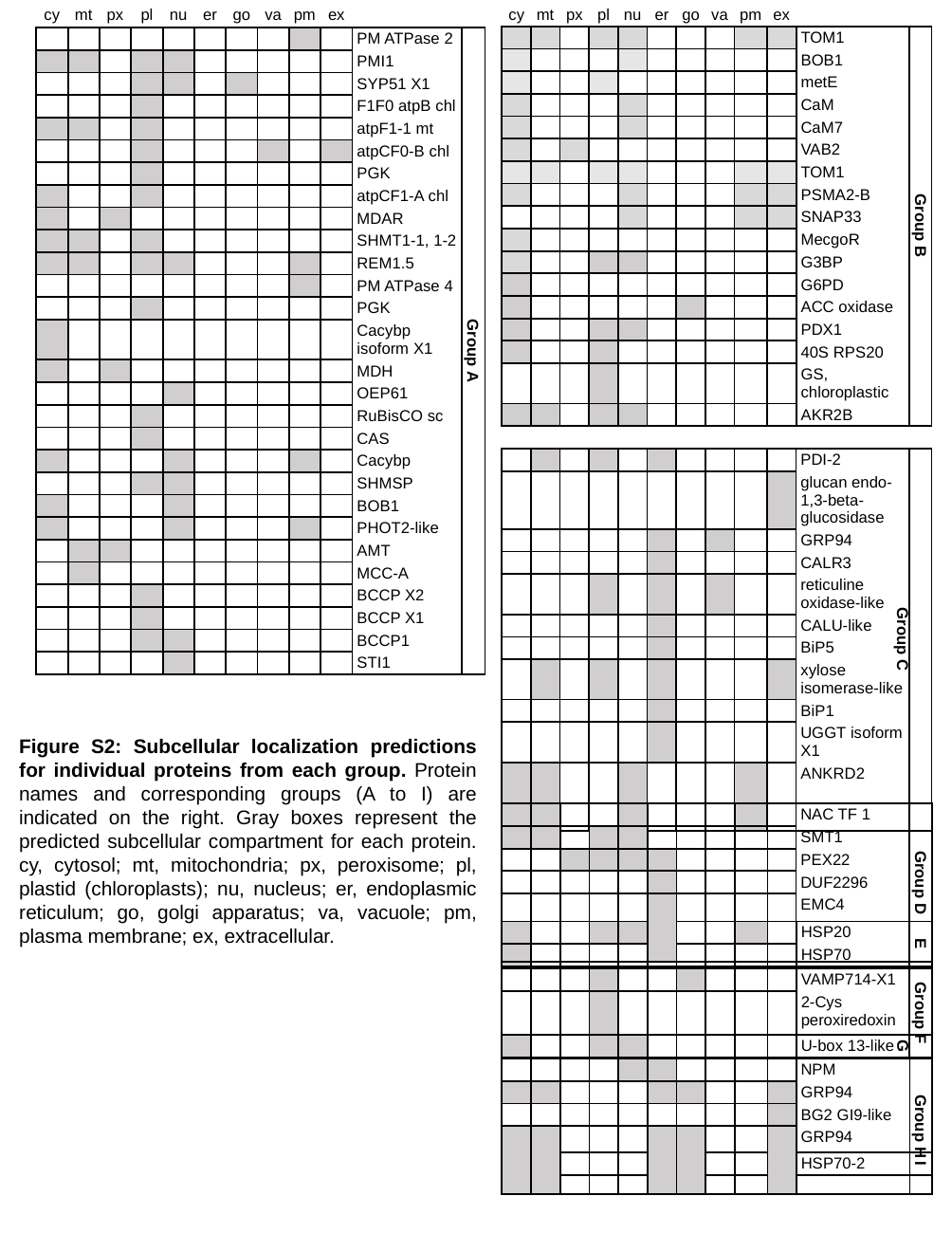

| cy | mt | px | pl | nu | er | go | va | pm | ex | | |
| --- | --- | --- | --- | --- | --- | --- | --- | --- | --- | --- | --- |
| | | | | | | | | | | PM ATPase 2 | Group A |
| | | | | | | | | | | PMI1 | |
| | | | | | | | | | | SYP51 X1 | |
| | | | | | | | | | | F1F0 atpB chl | |
| | | | | | | | | | | atpF1-1 mt | |
| | | | | | | | | | | atpCF0-B chl | |
| | | | | | | | | | | PGK | |
| | | | | | | | | | | atpCF1-A chl | |
| | | | | | | | | | | MDAR | |
| | | | | | | | | | | SHMT1-1, 1-2 | |
| | | | | | | | | | | REM1.5 | |
| | | | | | | | | | | PM ATPase 4 | |
| | | | | | | | | | | PGK | |
| | | | | | | | | | | Cacybp isoform X1 | |
| | | | | | | | | | | MDH | |
| | | | | | | | | | | OEP61 | |
| | | | | | | | | | | RuBisCO sc | |
| | | | | | | | | | | CAS | |
| | | | | | | | | | | Cacybp | |
| | | | | | | | | | | SHMSP | |
| | | | | | | | | | | BOB1 | |
| | | | | | | | | | | PHOT2-like | |
| | | | | | | | | | | AMT | |
| | | | | | | | | | | MCC-A | |
| | | | | | | | | | | BCCP X2 | |
| | | | | | | | | | | BCCP X1 | |
| | | | | | | | | | | BCCP1 | |
| | | | | | | | | | | STI1 | |
| cy | mt | px | pl | nu | er | go | va | pm | ex | | |
| --- | --- | --- | --- | --- | --- | --- | --- | --- | --- | --- | --- |
| | | | | | | | | | | TOM1 | Group B |
| | | | | | | | | | | BOB1 | |
| | | | | | | | | | | metE | |
| | | | | | | | | | | CaM | |
| | | | | | | | | | | CaM7 | |
| | | | | | | | | | | VAB2 | |
| | | | | | | | | | | TOM1 | |
| | | | | | | | | | | PSMA2-B | |
| | | | | | | | | | | SNAP33 | |
| | | | | | | | | | | MecgoR | |
| | | | | | | | | | | G3BP | |
| | | | | | | | | | | G6PD | |
| | | | | | | | | | | ACC oxidase | |
| | | | | | | | | | | PDX1 | |
| | | | | | | | | | | 40S RPS20 | |
| | | | | | | | | | | GS, chloroplastic | |
| | | | | | | | | | | AKR2B | |
| | | | | | | | | | | PDI-2 | Group C |
| --- | --- | --- | --- | --- | --- | --- | --- | --- | --- | --- | --- |
| | | | | | | | | | | glucan endo-1,3-beta-glucosidase | |
| | | | | | | | | | | GRP94 | |
| | | | | | | | | | | CALR3 | |
| | | | | | | | | | | reticuline oxidase-like | |
| | | | | | | | | | | CALU-like | |
| | | | | | | | | | | BiP5 | |
| | | | | | | | | | | xylose isomerase-like | |
| | | | | | | | | | | BiP1 | |
| | | | | | | | | | | UGGT isoform X1 | |
| | | | | | | | | | | ANKRD2 | |
Figure S2: Subcellular localization predictions for individual proteins from each group. Protein names and corresponding groups (A to I) are indicated on the right. Gray boxes represent the predicted subcellular compartment for each protein. cy, cytosol; mt, mitochondria; px, peroxisome; pl, plastid (chloroplasts); nu, nucleus; er, endoplasmic reticulum; go, golgi apparatus; va, vacuole; pm, plasma membrane; ex, extracellular.
| | | | | | | | | | | NAC TF 1 | Group D |
| --- | --- | --- | --- | --- | --- | --- | --- | --- | --- | --- | --- |
| | | | | | | | | | | SMT1 | |
| | | | | | | | | | | PEX22 | |
| | | | | | | | | | | DUF2296 | |
| | | | | | | | | | | EMC4 | |
| | | | | | | | | | | HSP20 | E |
| --- | --- | --- | --- | --- | --- | --- | --- | --- | --- | --- | --- |
| | | | | | | | | | | HSP70 | |
| | | | | | | | | | | VAMP714-X1 | Group F |
| --- | --- | --- | --- | --- | --- | --- | --- | --- | --- | --- | --- |
| | | | | | | | | | | 2-Cys peroxiredoxin | |
| | | | | | | | | | | U-box 13-like | G |
| --- | --- | --- | --- | --- | --- | --- | --- | --- | --- | --- | --- |
| | | | | | | | | | | NPM | Group H |
| --- | --- | --- | --- | --- | --- | --- | --- | --- | --- | --- | --- |
| | | | | | | | | | | GRP94 | |
| | | | | | | | | | | BG2 GI9-like | |
| | | | | | | | | | | GRP94 | |
| | | | | | | | | | | HSP70-2 | I |
| --- | --- | --- | --- | --- | --- | --- | --- | --- | --- | --- | --- |
