# Supplementary material for: Proximity Labelling‐Based Proteomics Identifies Antiviral Host Factors Associated With the Potexvirus Replicase
Source: Mol Plant Pathol. 2026 Mar 19;27(3):e70239. doi: 10.1111/mpp.70239 (PMC13097338; doi:10.1111/mpp.70239)
Supplement: Supplementary file 6 — Table S1: The target gene fragments for construction of host factors knockdown. [file MPP-27-e70239-s008.docx]

Table S1. The target gene fragments for construction of host factors knockdown

| Gene | Sequences (5’-3’) |
| --- | --- |
| NbCAS | AGGCAGGAGAGGAAGTTTTGAAGAATGCTTCTCCTGTCATATCTGACGCCACTAAGAAAGCC  CAAGAGGCAATGCAGAGCGCTGGCATGGACGCTCAACCAGTGATGACTGCAGCCAAGACAGTTGTTGATGCAGCTCAACAGACATCCAAGGTGATTGAAGGGGCCAAACCAATCGCCTCATCTACAGTTGAAAC |
| NbREM1.5 | ATCTCAAGAAGCAGCTGTTGATAATTCTCCTGCTGCCATGGCTACCAAAGCTGATGATTCTAA  AGCTCTCGCCACTGTTCCTCCACCAAAGACTGATTCTTCAACAAAGAAGAGTTCAAAGGGATCCCTCGATAGAGACATTGCTCTCGCACACCTTGAAACAGAGAGAAGGAATTCTTATAT |
| NbCBP | TCGTAACTTGGAGAAGCTGTCAAAAGACGGTGCATCAGCGCCATCTTCGCAAATACCAGCTC  CTGTTTCAACTGCCGCAAAGGTGACCCCTAGCACATTTCTGAACTATGTCTCTGTTGCATCATTCAGTTGGGATCAAGACAATGATAAAGTGAAGATTTATCTCTCTTTGGAAGGAGTCGATCAGGAGAAAGCGGAGA |
