# Supplementary material for: Proximity Labelling‐Based Proteomics Identifies Antiviral Host Factors Associated With the Potexvirus Replicase
Source: Mol Plant Pathol. 2026 Mar 19;27(3):e70239. doi: 10.1111/mpp.70239 (PMC13097338; doi:10.1111/mpp.70239)
Supplement: Supplementary file 7 — Table S2: The synthetic DNA fragment encoding 27 amino acids of cytochrome P450 2C1. [file MPP-27-e70239-s003.docx]

Table S2. The synthetic DNA fragment encoding 27 amino acids of cytochrome P450 2C1

| Gene | Accession number | Sequence (5’-3’) |
| --- | --- | --- |
| cytochrome P450 2C1 (1-27 aa) | Addgene, cat. no. 79055 | ATGGATCCTGTTGTGGTTCTTGGGTTGTGTCTCTCATGCTTGCTCTTACTG  TCCCTATGGAAGCAAAGCTATGGTGGTGGA |
