# Supplementary material for: Proximity Labelling‐Based Proteomics Identifies Antiviral Host Factors Associated With the Potexvirus Replicase
Source: Mol Plant Pathol. 2026 Mar 19;27(3):e70239. doi: 10.1111/mpp.70239 (PMC13097338; doi:10.1111/mpp.70239)
Supplement: Supplementary file 8 — Table S3: cDNA sequence used for construction of transient overexpression of host factors. [file MPP-27-e70239-s006.docx]

Table S3. cDNA sequence used for construction of transient overexpression of host factors.

| Gene | Accession number | Sequence (5’-3’) |
| --- | --- | --- |
| NbCAS | AB720027.1 | ATGGCGCTTAGAGCTTCAGCCACCGTAAAATCACATCTTCCTCCGCCTTCTTC  TTCACCTATTAAAATCTTTAATTTCCCTACACTCTCCCAGAAACCGGAATTCACTTCAAATTCTGTGTCAGTATCACTCTCTACTTCAACTGCTCTTTTCCTATTTCCTCTTTTCACTGCTACCCATGAAGCCAGAGCACTCAGCTTACCCAAGGAAGATATTGTCTCTTCCCTTAATCAGGTAGAATCTGCAGTTAATCAAGCTCAAGAGGTTGGTTCGAACATCTTTGATGCTGCAAGCCGAGTGATTGGGCCCGTGATTGAATTTGTAAAGCCCGGGATTGATGTGGCATTGCCTTTAGTAAAGCAGGCAGGAGAGGAAGTTTTGAAGAATGCTTCTCCTGTCATATCTGACGCCACTAAGAAAGCCCAAGAGGCAATGCAGAGCGCTGGCATGGACGCTCAACCAGTGATGACTGCAGCCAAGACAGTTGTTGATGCAGCTCAACAGACATCCAAGGTGATTGAAGGGGCCAAACCAATCGCCTCATCTACAGTTGAAACTATTTCATCAGCTGATCCAGCTATTATTGCAGTGGCTGGTGGCACATTATTCCTGGCATATCTTCTATTTCCCCCTGTCCTCTCCGCTCTCTCTTTCAGCCTTCGTGGCTACAAGGGTGAACTAACTCCTGCTCAGACACTGGACCAGATGTGTTCTAAGAATTATGTCTTGATTGATATTAGAACAGAGAAGGACAAGGATAAGGCTGGAATTCCTCGTCTTCCATCTAGTGCTAAAAACAAGATGATTCAAATCCCTTTGGAAGATCTACCGAGCAAACTAAGGAGTCTCGTGAGAAATGCGAAGAAAGTGGAAGCTGAATTAGTAGCTCTGAAGATATCATACCTCAAGAAAATCAACAAAGGGTCTAACATTGTGATAATGGACTCGTACTCTGATTCAGCTAAAACAGTTGCTAAAACACTGACGAGCCTTGGCTTTAAGAACTGCTGGATCATGACTGATGGCTTCTCTGGAGGGAGGGGTTGGTTGCAGAGTAAGCTGGGAACAGATTCTTACAACTTTTCTTTTGCCCAAGTCTTATCACCATCAAGAGTCATACCAGCAGCAGCTAGACGTTTTGGTACAACAGGCACTGTCAAATTGCTTTCAGGGGGTAGTGAT |
| NbREM1.5 | ON392762.1 | ATGGCAGAAGCAACTCCAGTATCTCAAGAAGCAGCTGTTGATAATTCTCCTGCTGCCATGGCTACCAAAGCTGATGATTCTAAAGCTCTCGCCACTGTTCCTCCACCAAAGACTGATTCTTCAACAAAGAAGAGTTCAAAGGGATCCCTCGATAGAGACATTGCTCTCGCACACCTTGAAACAGAGAGAAGGAATTCTTATATTAAGGCATGGGAAGAAAGTGAAAAAAGCAAGGTGGAAAACAAGGCCGAAAAGAAGCTCTCTGCAGTTGGGACATGGGAGAACACCAAGAAAGCAAATCTTGAAGCTAAACTGAAGAAACTTGAGGAGCAACTAGAAGAAAAGAAAGCAGAATATGCGGAGAAGATTAAAAATAGAGTAGCCGCAGTTCACAAGGAGGCTGACGAAAAGAGAGCTATGGTTGAAGCCAGAAAGGGAGAAGAACTTCTTAAAGCAGATGAGATGGCTGCCAAGTATCGCGCCACCGGACAAGCCCCTAAGAAGTTGCTTGGATGCCTTGGATGC |
| NbCBP | Nbe.v1.1.chr07g10030 | ATGGCAGCAAGCGATTTATCTTTGGATTTGGAGGAACTTAAGCATCTTCTGAG  CATAGCGAAACGTCCTCGTGTTGTTTCTCTCATTTCCTCTGAGATTCGTAACTTGGAGAAGCTGTCAAAAGACGGTGCATCAGCGCCATCTTCGCAAATACCAGCTCCTGTTTCAACTGCCGCAAAGGTGACCCCTAGCACATTTCTGAACTATGTCTCTGTTGCATCATTCAGTTGGGATCAAGACAATGATAAAGTGAAGATTTATCTCTCTTTGGAAGGAGTCGATCAGGAGAAAGCGGAGACCGAGTTCAAGCCTATGTCATTTGATGCTAAGTTCCACGATGTACACGGGAAGAACTTCCGCTTCTCTTTACCAAAATTGAACAAAGAGATTGTACCTGAGAAATGTAAGGTGCTTGTGAAACCCACGAGGGTTGTTATCACCTTGATCAAGGCCTCCAAAGGAAACTGGTTGGATCTGCATTACAAAGAGGATAAGTTCAAGCCAAATTTGGACAAAGAAAAAGACCCCATGGCTGGAATTATGGATTTGATGAAGAACATGTATGAGGACGGTGATGAAGAGATGAAACGGACTATTGCAAAAGCTTGGACTGATGCAAGATCTGGCAAGGCAGCTGACCCGTTGAAGAGATTCAGT |
