# Supplementary material for: Proximity Labelling‐Based Proteomics Identifies Antiviral Host Factors Associated With the Potexvirus Replicase
Source: Mol Plant Pathol. 2026 Mar 19;27(3):e70239. doi: 10.1111/mpp.70239 (PMC13097338; doi:10.1111/mpp.70239)
Supplement: Supplementary file 9 — Table S4: Primers used in this study. [file MPP-27-e70239-s001.docx]

Table S4. primers used in this study.

| Primer name | Sequence (5’-3’) |
| --- | --- |
| pCAMBIA25-F | CGGGGGACTCTTGACCATGGTCGAC |
| GFP-SpeI-Turbo-R | ACAGTATTGTCTTTACTAGTCTTGTACAGCTCGTCCATGCCG |
| pC1301-1-SalI-ER-GFP5’-F | CTTGACCATGGTCGACATGAAGGTACAGGAGGGTTTGT |
| pC1301-1-ERGFP5-SpeI-Turbo-R | ACAGTATTGTCTTTACTAGTAGATCTGTATAGTTCATCCATGCC |
| pCAMBIA-MET-F | GGACTCTTGACCATGGTCGACATGTCAAACGTTCGGAACG |
| MET-SpeI-Turbo-R | TGTCTTTACTAGTGGGAAATTCCAAGGTGATGGG |
| MET-SpeI-Turbo-F | ATTTCCCACTAGTAAAGACAATACTGTGCCTCTG |
| Turbo-myc-pCAMBIA-R | GTCACCCTAGGTACCGGATCCCAGGTCCTCTTCTGAGATGAGTTTTTGTT  CCTTTTCGGCAGACCGC |
| pC1301-1-GFP5-SpeI-Turbo-F | TGAACTATACAGATCTACTAGTAAAGACAATACTGTGCCTCTG |
| pCAM-GFP-Turbo2-myc-HDEL-R | TTACAGCTCGTCATGCTCGCTTGCGGCCCCATTCAGGTCCTCTTCTGAGA  TGAGTTTTTGTTCCTTTT |
| Turbo-myc-HDEL-pCAM-R2 | CCTAGGTACCGGATCCTTACAGCTCGTCATGCTCGC |
| 2C1-31aa-GFP-F | ATGGTGAGCAAGGGCGAGGAGCT |
| pCAM-MET-GFP-F | GGAATTTCCCACTAGTGTGAGCAAGGGCGA |
| pCAM-MET-GFP-Turbo-myc-R | CCTAGGTACCGGATCCCAGGTCCTCTTCTG |
| pCAMBIA-SalI-NbCAS-F | CTTGACCATGGTCGACATGGCGCTTAGAGCTTCAGC |
| pCAM-NbCAS-SpeI-R-new | CCTTGCTCACACTAGTATCACTACCCCCTGAAAGCAATTTG |
| pCAM-calcyclin-F | TTGACCATGGTCGACATGGCAGCAAGCGATTTATCTTTGG |
| calcyclin-pCAM-R | GCTCACCATACTAGTTCAACTGAATCTCTTCAACGGGTC |
| pCAM-NbREM1.5-F | TTGACCATGGTCGACATGGCAGAAGCAACTCCAGTATCTC |
| NbREM1.5-pCAM-3R | CTTGCTCACACTAGTGCATCCAAGGCATCCAAGCAAAC |
| pTV-SpeI-NbCAS-F | GATCCTAGAACTAGTGTTTCAACTGTAGATGAGGCGATTGG |
| pTV-NbCAS-KpnI-R | GGCGAATTGGGTACCAGGCAGGAGAGGAAGTTTTGAAGAA |
| NbREM1.5-pTV-F | GATCCTAGAACTAGTATATAAGAATTCCTTCTCTCTGTTTC |
| pTV-NbREM1.5-R | GGCGAATTGGGTACCATCTCAAGAAGCAGCTGTTG |
| NbCBP-pTV-F | GATCCTAGAACTAGTTCTCCGCTTTCTCCTGATCGACTC |
| pTV-NbCBP-R | GGCGAATTGGGTACCTCGTAACTTGGAGAAGCTGTCAAAAG |
| qPCR-NbCAS-1F | TATTGCAGTGGCTGGTGGCA |
| qPCR-NbCAS-1R | TGGTCCAGTGTCTGAGCAGG |
| qPCR-NbREM1.5-3F | AGCTCTCTGCAGTTGGGACA |
| qPCR-NbREM1.5-3R | CCTCCTTGTGAACTGCGGCT |
| qPCR-NbCBP-2F | TCTGAGCATAGCGAAACGTCC |
| qPCR-NbCBP-2R | GCGCTGATGCACCGTCTTTT |
| PlAMV3877F | CCTCATTCTCCCTGCTGAAG |
| PlAMV4010R | CTTGAGGGGGTCTTTGATGA |
| NbPP2A-F | GACCCTGATGTTGATGTTCGCT |
| NbPP2A-R | GAGGGATTTGAAGAGAGATTTC |
| NbREM1.5/GW/F01 | AAAAAGCAGGCTACCATGGCAGAAGCAACTCCAGT |
| NbREM1.5/GW/R01 | AGAAAGCTGGGTTGCATCCAAGGCATCCAAGCAAC |
| attB1 adaptor primer | GGGGACAAGTTTGTACAAAAAAGCAGGCT |
| attB2 adaptor primer | GGGGACCACTTTGTACAAGAAAGCTGGGT |
